# A Study of Fluid Intake, Hydration Status, and Body Composition of Pregnant Women in Their Third Trimester, and Relationships with Their Infant’s Birth Weight in China: A Prospective Cohort Study

**DOI:** 10.3390/nu16070972

**Published:** 2024-03-27

**Authors:** Yongye Song, Fan Zhang, Xing Wang, Guotian Lin, Limin He, Zhixiong Lin, Na Zhang, Guansheng Ma

**Affiliations:** 1 Department of Nutrition and Food Hygiene, School of Public Health, Peking University, 38 Xue Yuan Road, Haidian District, Beijing 100191, China; songyongye@bjmu.edu.cn (Y.S.); wangxing_98@126.com (X.W.); mags@bjmu.edu.cn (G.M.); 2 Laboratory of Toxicological Research and Risk Assessment for Food Safety, Peking University, 38 Xue Yuan Road, Haidian District, Beijing 100191, China; 3 International School of Public Health and One Health, Hainan Medical University, 3 Xue Yuan Road, Longhua District, Haikou 571199, China; zhangfan@hainmc.edu.cn (F.Z.); banxia75@163.com (L.H.); 4 School of Health Medicine, University of Sanya, 191 Xue Yuan Road, Jiyang District, Sanya 572022, China; linguotian123@163.com; 5 Haikou Hospital of the Maternal and Child Health, 6 Wen Tan Road, Guo Xing Avenue, Qiongshan District, Haikou 570203, China; zgyglzx@163.com

**Keywords:** fluid intake, hydration status, body composition, pregnant women, birth weight

## Abstract

Background: Water intake and hydration status may potentially influence maternal and child health. However, there is little research regarding this topic. Objectives: This study aimed to investigate pregnant women’s total fluid intake (TFI) levels, hydration status, and body composition and further explore their relationship with infant birth weight. Methods: A 7-day, 24 h fluid intake recorded was applied to determine participants’ TFI levels. Morning urine samples were collected and tested to evaluate their hydration status. Maternal body compositions in their third trimester and infant birth weights were measured. Results: A total of 380 participants completed the study. The TFI was insufficient for pregnant women during their third trimester (median = 1574 mL), with only 12.1% of participants meeting the recommended adequate fluid intake level for pregnant women living in China (1.7 L per day). With the increasing TFI values, the urine osmolality decreased, which showed statistical significance among the four groups (χ^2^ = 22.637, *p* < 0.05). The participants displayed a poor hydration status. Meanwhile, the percentage of participants who were in dehydrated status decreased (χ^2^ = 67.618, *p* < 0.05), while body water content and basal metabolic rate increased with the increase in TFI levels (χ^2^ = 20.784, *p* < 0.05; χ^2^ = 14.026, *p* < 0.05). There were positive linear relationships between plain water intake, the basal metabolic rate of pregnant women and their infant birth weight (SE = 0.153, *p* < 0.05; SE = 0.076, *p* < 0.05). Conclusions: Water intake was insufficient, and poor hydration status was common among pregnant women in China. There may be potential relationships between plain water intake, basal metabolic rate, and infant birth weight.

## 1. Introduction

Water accounts for approximately 60~70% of a healthy adult’s body weight [1]. It exerts an enormous function in maintaining electrolyte homeostasis, retaining stable body temperature, and lubricating organs, joints, muscles, and tissues [2,3]. There exist three pathways for water input: water intake from beverages, water intake from food, and endogenous water. The four ways of water elimination from the body refer to water excreted through the kidneys in the form of urine, water excreted through skin evaporation in the form of sweat, water excreted through pulmonary respiration, and water excreted through the intestine in the form of feces [4,5]. As a result, the daily intake and discharge of water are maintained at around 2500 mL. Hydration status is linked to the balance of water input and water elimination in the human body [4]. Pregnancy is a special and complex period during which women are subjected to anatomical and physiological changes. It is of great importance to meet the increased maternal metabolic demands. Apart from that, it is essential for meeting the requirements of fetal development [6]. Growing evidence has demonstrated that maternal nutrition has a direct effect on body composition and weight at birth. In addition, it has a long-lasting influence on health status and metabolic responses in adulthood [7,8].

During pregnancy, blood volume significantly increases [9]. In addition, oxygen consumption increases by 30%, metabolic rate by 15%, and tidal volume by 30~50% [10,11]. Additionally, an increase occurs in the glomerular filtration rate (GFR) and effective renal plasma flow (RPF) [12]. As a result, total body water content roughly increases by 6.5~8 L [6,13]. Body water distribution during pregnancy affects the hydration status, as a study showed that, except for pregnancy elements (placenta, infant, and uterus), the added hydration is mainly extracellular [14]. Additionally, human chorionic gonadotropin (HCG) levels increase during pregnancy, leading to changes in water homeostasis and osmolality [15]. They may also be related to regulatory system changes and hormone-level fluctuations [16]. Plasma osmolality decreases during pregnancy, and changes also occur in the osmolality threshold for vasopressin release [17]. This leads to increased activity in sympathetic and renin–angiotensin–aldosterone pathways, which is critical for water homeostasis [18]. A significant increase in the plasma vasopressin enzyme may explain the changes in water homeostasis in the third trimester of pregnancy [16]. In addition, body water distribution will be affected, as the extracellular and plasma volume of pregnant women increased by 50~70%, accompanied by significant accumulation of sodium retention shared by the mother and fetus. During pregnancy, a water imbalance can even predict gestational hypertension [19], pre-eclampsia [20], low birth weight, and poor pregnancy outcomes [21,22].

Total water intake consists of two parts, namely, the fluid intake from water and beverages (accounting for approximately 50%) and the water intake from food (accounting for approximately 40%) [1]. Daily TFI is defined as the amount of water and beverages consumed, with water from food being excluded. The recommended adequate fluid intake level for pregnant women in China is 1.7 L per day (excluding water intake from food) [23]. However, previous studies have found that women during pregnancy generally suffer from insufficient fluid intake. A water intake investigation was conducted on 300 pregnant Indonesian women using the 7-day, 24 h fluid intake record. The results showed that approximately 42% of participants were under Indonesia’s recommended adequate fluid intake level (2048 mL/d) [24]. A previous study found that the median TFI level among Chinese pregnant women in the second trimester was 1485 mL [25]. Thus, the fluid intake of women needs to be improved during their pregnancy.

A low birth weight refers to a newborn weighing less than 2500 g [26]. A large prospective cohort study in China showed that the low-birth-weight incidence rate was 14.5% [27]. Low birth weight is a key determinant of the health status and development of newborns, which can increase the morbidity and mortality of newborns. It is also closely related to the risk of chronic diseases, including hypertension, diabetes, obesity, and cardiovascular and cerebrovascular diseases in adulthood [28,29]. A study was carried out in Canada among 196 pairs of mothers and live singleton newborns whose gestational ages were 37 weeks or more. The body composition of 196 women between 4 and 12 h postpartum was measured. In this study, compared with the mother’s gestational weight gain, total body water content was a major determinant of birth weight variability [22]. A prospective study conducted in America followed 105 healthy women who delivered of-term infants [30]. Body composition was evaluated eight times during their pregnancy. The result showed that body water content was considered an independent predictor of infant birth weight. A case-control study performed in America indicated that fluid intake was associated with low birth weight. Women with lower fluid intake levels during pregnancy developed lower birth weights of their infants [31]. Twenty-nine pregnant women and their full-term infants were followed in a longitudinal study in Poland. However, the result showed no significant correlation between maternal water intake and infant birth weight [18]. The nutrition required during different trimesters of pregnancy is not invariable, but it is a dynamic process. Conclusions regarding body composition, particularly total body water, are still inconsistent in the literature [19,20,32]. Nevertheless, abnormal fluid might be the cause of maternal and fetal pathologies, including intrauterine growth restriction or hypertensive complications during pregnancy [33,34].

Therefore, the aims of this study were the following: (1) to determine and assess the TFI levels and body compositions of women in their third trimester of pregnancy; (2) to assess the urine biomarkers and evaluate the hydration status; and (3) to explore the relationships between maternal fluid intake, body composition, and infant birth weight. The results can provide accurate and meaningful references for promoting the fluid intake level for pregnant Chinese women in their third trimester. Moreover, these can provide possible ways to improve infants’ birth weights.

## 2. Materials and Methods

### 2.1. Study Design

A convenience sampling method was adopted in this study. Recruitment was performed according to the inclusion and exclusion criteria. Pregnant women who attended outpatient clinics at the Haikou Maternal and Child Health Hospital from August 2020 to March 2021 were enrolled.

### 2.2. Sample-Size Calculation

The variable used for the sample-size calculation was the incidence of low birth weight. The relevant study in China reported that the incidence of low birth weight was 14.5% [27]. The sample size was determined on the basis of the following formula: n = *Z*_1−α/2_^2^*p*(1 − *p*)/*e*^2^. In the formula, we set α = 0.05 and Z_1−α/2_ = 1.96. Moreover, “e” was the error bound and was set as 4%. The required sample size was 298. Considering a dropout rate of 20%, 372 participants need to be recruited.

### 2.3. Participants

The criteria for inclusion in the study were as follows: first, maternity screening conducted before 28 weeks gestation; being aged between 21 and 35 years old; a singleton pregnancy; being in a good health condition before enrollment; routine prenatal examinations at the hospital where the study was conducted (Haikou Hospital of The Maternal and Child Health); and having the ability to complete questionnaires independently. The following exclusion criteria were applied: smoker; habitual alcohol consumer (>20 g/day) [35]; any fluid intake intervention; engagement in rigorous physical activity; or the presence of endocrine diseases, urinary system diseases, digestive system diseases, cardiovascular diseases, or cognitive disorders; diabetes mellitus; or any other pre-existing diseases before pregnancy.

### 2.4. Ethics

The study protocol underwent a thorough review and get approval by the Ethical Review Committee of the Hainan Medical University (with an identification code of 2018-4). The study protocol has been registered on the Chinese Clinical Trial Registry website under the trial registration number Chi CTR 800019284. The study adhered to the principles of the Declaration of Helsinki. Participants were informed of the background, purpose, duration, steps, and potential issues at the beginning of the study.

### 2.5. Study Procedure

This prospective cohort study was carried out from August 2020 to March 2021. Maternal socio-economics, socio-demographics, and other basic information, including the participants’ pregnancy, childbearing, disease, and drug-usage histories, was collected after recruitment. Meanwhile, participants’ heights, weights, and body compositions were measured in the third trimester of pregnancy. The 7-day, 24 h fluid intake record was used to observe the fluid intake patterns of these participants in their third trimester. Their first morning urine samples were collected by themselves. The samples were tested within 2 h by professional laboratory technicians on day 4 during the 7 consecutive days. Infant birth weight was measured within 1 h after delivery. The temperature and humidity data for this period were acquired from the China Meteorological Administration statement. The timeline for study duration, data collection, and collected indicators is presented (Table 1).

### 2.6. Physical Measurements

For height and weight, investigators were uniformly trained prior to the study. Participants were measured using uniform instruments (DHM-300; Huaju, Yiwu, Zhejiang, China). The values were measured twice according to the standardized method. Height values were accurate to 0.1 cm and weight values were accurate to 0.1 kg, respectively (BMI is calculated as weight (kg)/height squared (m^2^)). The average values were calculated and reported.

### 2.7. Tests of Blood Pressure and Blood Glucose

For blood pressure measurements, an electronic upper-arm sphygmomanometer (U10L; Omron, Dalian, China) was used by trained investigators to measure the participants’ blood pressure. The standard process was used for measurement in this study [36]. Participants were asked not to perform intense exercise for 1 h before the measurement and to stay calm for 5 min. Eating or drinking beverages containing caffeine was also not allowed. They were asked to remove heavy clothing and sit in an upright and relaxing position. For blood pressure measurement, a cuff was wrapped around the participant’s left arm 1 to 2 cm above the medial joint of the elbow. The readings of blood pressure were accurate to 2 mmHg. Two consecutive measurements were taken, and the average values of both the systolic and diastolic blood pressure measurements were recorded.

For blood glucose, the participants’ blood glucose was determined with elbow venous blood using an automatic biochemical analyzer (AU5800, Beckman, Brea, CA, USA) by laboratory physicians. Blood glucose levels during the fasting state of the participants were measured in the study.

### 2.8. Body-Composition Measurements

Participants’ body compositions were measured by uniformly trained investigators using a human-fat-measuring instrument (BC-601; TANITA; Tokyo, Japan). Bioelectrical impedance analysis (BIA) was the instrument’s method. Impedances at different segments were measured, including the right arm, left arm, right leg, left leg, and trunk for all frequencies. Due to the different electrical characteristics of body tissues, the volume of conductive tissues was determined according to the electrical resistances of various tissue parts of the human body. Thus, the body composition was estimated. Participants were required to discharge their night soil and urine before measurements and wear lightweight clothing, with their socks, shoes, and any metal jewelry removed.

In the meantime, it is necessary for them to be prepared with clean soles. Information was imported into the instrument by investigators, such as the participants’ ages, genders, and heights. When the value on the screen was reset to zero, participants were instructed to stand on the instrument. Their palms and soles contacted the electrode surface. Their torsos and upper limbs were maintained at an angle of approximately 30°. Participants were required to remain quiet and not move during the measurement process. The results were displayed on the screen automatically when the measurements were finished. The results included data on body water content, bone mineral content, percent of body fat, skeletal muscles, and basal metabolic rate.

### 2.9. Assessment of Daily Total Fluid Intake (TFI)

Daily total water intake levels refer to the sum of daily total fluid intake (TFI) and daily water intake from food. TFI refers to the fluid intake level from water and beverages, with water from food excluded. The amount of water intake from food was not assessed in the present study.

A “7-day 24-h fluid intake record” was applied to observe the fluid intake level [37,38,39,40]. This record has been validated and employed in many studies, which are authoritatively and widely applied to record fluid intake in various countries [41,42,43]. Expert consensus has been reached, as the record has been subjected to expert consultation and repeated argumentation [40,44]. Fluid intake types were classified according to the classification criteria [45]. The included types were plain water, dairy products, and sugar-sweetened beverages. Plain water refers to tap, packaged, mineral, and purified water. Dairy products included pure milk, yogurt, and other dairy products with no sugar added during the production. Sugar-sweetened beverages (SSBs) refer to beverages with the addition of sugar during production, including carbonated, fruit and vegetable juice, protein, sugary coffee, plant-based, flavored, and special-purpose beverages. Each participant was provided with a uniformly customized cup. A cup with a scale was used to estimate the amount of fluid intake each time. Fluid intake levels and types for 7 consecutive days were recorded in detail. The capacity of the cup was 400 mL, with the cup scale accurate to 10 mL.

### 2.10. Tests Conducted for Urine Biomarkers

For urine osmolality, values were achieved from participants’ first morning urine. The samples were collected in this study to test urine biomarkers. Sterile disposable urine-sample cups were used for urine collection. Urine osmolality was measured by an osmotic pressure molar concentration meter (SMC 30C; Tianhe, Tianjin, China) with the freezing-point method. The process of the test was in accordance with the Standard Operating Procedure. Urine osmolality values were then applied to evaluate the hydration status of the participants.

For urine specific gravity (USG), it was tested with an automatic urinary sediment analyzer (FUS-200; Dirui; Changchun, China). The uric dry-chemistry method was applied to urine testing, and color development was achieved by replacing hydrogen ions with cations. The urine samples were delivered to the laboratory and tested within 2 h of collection.

### 2.11. Evaluation and Definition of Hydration Status

With reference to current studies [38,46], urine osmolality was used for the assessment and classification of participants’ hydration status. Based on urine osmolality values, participants were categorized into three groups in terms of their hydration status: groups with a dehydrated status, normal hydrated status, and optimal hydrated status. The classification of hydration status determination using urine osmolality is presented below (Table 2).

### 2.12. Measurement of Infant Birth Weight

Infant birth weight was measured within 1 h after delivery. The measurement was taken using a weight-measuring device (HLZ-20; Hualizheng, Tianjin, China) by professional obstetricians while the infant wore light clothing. The measurement value was accurate to within 0.1 g.

### 2.13. Temperature and Humidity

The present study was implemented in Haikou city. Daily minimum and maximum temperatures were recorded during the 7 consecutive days. The data were provided by the Meteorological Administration of China. The median temperature was considered as the day’s temperature, and the average temperature over the seven days was calculated. What is more, humidity was recorded during the study period.

### 2.14. Quality Control

A unified procedure was developed, and all the investigators were uniformly trained before the study. A research guide was designed, including the research protocol, questionnaire, methodology, and timeline, and sample and indicators to be collected. The reasons for and the time for dropping out were also collected in a comprehensive way. During the entire research process, all procedures were subjected to strict supervision by quality control staff. Trained investigators guided participants to complete the questionnaire. Double checks were performed on the completeness and logicality of the questionnaires. If any errors were found, the participants were contacted to correct the records until they met the requirements. Prior to data entry, each item in the questionnaire or record was verified, and the incorrect records were deleted.

### 2.15. Statistical Methods

Data entry was completed using the EpiData 3.1 software. This process was performed using the double-entry method and the database was created. The SPSS Statistics 26.0 (IBM Crop., Armok, NC, USA) was applied for statistical analysis. The continuous variables were subjected to normality tests. The medians (M) were used to report and analyze abnormal distribution data. The interquartile ranges of the interval limited by the 25th and 75th percentiles (Q_1_~Q_3_) were also shown. A one-way ANOVA was used to compare differences in the normally distributed data among the four groups. The Kruskal–Wallis H test was applied for comparison of differences in the abnormal distribution data. The proportions of participants meeting the adequate fluid intake (AI) level in China, fluid intake amount and percent, and hydration status among the four groups were compared by chi-square test. Multiple comparisons were performed using the Student–Newman–Keuls (SNK) method (*p* < 0.05). The Spearman correlation was used to test the correlations between total drinking and plain water intake. Adjusted analyses were carried out using multivariable linear regression models to analyze the linear relationships between different types of fluid intake and infant birth weight. All tests were two-sided, and *p* < 0.05 was considered statistically significant.

## 3. Results

### 3.1. Participants’ Characteristics and the Environment

In total, 380 pregnant women who were in compliance with the inclusion criteria were recruited for our study. Finally, 380 participants completed the study, resulting in a completion rate of 100%. More detailed characteristics of the 380 participants are summarized in Table 3.

According to the quartiles of participants’ TFI levels, they were categorized into four groups, namely, the LFI_1_ (low fluid intake 1), LFI_2_ (low fluid intake 2), HFI_1_ (high fluid intake 1), and HFI_2_ (high fluid intake 2), (Q_1_: 1200~1487 mL, Q_2_: 1488~11573 mL, Q_3_: 1574~1641 mL, and Q_4_: 1642~1950 mL). Among the factors investigated, no significant differences in the factors of age, height, weight, and blood pressure were observed between the four groups (all *p* > 0.05). However, significant differences were observed between the BMIs among the four groups (F = 9.500, *p* < 0.05).

The average value of temperature was 27.6 ± 3.3 °C, with an average humidity of 78.3 ± 7.9% RH in Hainan during the study period.

### 3.2. Measurement of TFI of Participants with Different TFI Levels

Among the 380 participants, the median value of TFI was 1574 mL. Approximately 85.2% of the participants were below China’s recommendation for an adequate fluid intake level (1.7 L per day for pregnant women). The most common source for participants’ fluid intake was plain water, as it represented 94.3% of daily TFI. Dairy products were the second largest contributor to TFI, accounting for 4.1%. The median dairy product intake level was 59 mL. SSBs (sugar-sweetened beverages) accounted for a small proportion of TFI (Table 4).

### 3.3. Measurement of Urine Biomarkers of Participants with Different TFI Levels

The data indicated that the increase in TFIs decreased urine osmolality from the LFI_1_ to HFI_2_ groups and significantly differed between the four groups (χ^2^ = 22.637, *p* < 0.05). There were 15.0% of participants who were in an optimal hydration status in the third trimester when assessed by urine osmolality. The median USG value of the participants was 1.015.

Hydration status improved with the increase of TFI and differed significantly between the four groups (χ^2^ = 67.618, *p* < 0.05). USG, urine pH, urine creatinine, and uric acid values differed significantly between the four groups (χ^2^ = 19.092, *p* < 0.05; χ^2^ = 9.791, *p* < 0.05; χ^2^ = 5.939, *p* < 0.05; χ^2^ = 10.680, *p* < 0.05; and χ^2^ = 14.030, *p* < 0.05). No significant differences in urea values were observed between the four groups (*p* > 0.05) (Table 5).

### 3.4. Measurement of Body Compositions of Participants with Different TFI Levels

The basal metabolic rate and body water content differed significantly among the four groups (χ^2^ = 20.784, *p* < 0.05; χ^2^ = 14.026, *p* < 0.05). Participants with higher TFI levels had higher values of basal metabolic rate and body water content. No statistically significant differences were identified in the skeletal muscle, bone mineral content, or percent body fat (all *p* > 0.05) (Table 6).

### 3.5. Relationships between Fluid Intake Types, Maternal Body Composition, and Infant Birth Weight

The sample consisted of 380 singleton births in this study, with a mean birth weight of 3206 ± 342 g. The mean values of infant birth weight in the four groups from LFI_1_ to HFI_2_ were 3231 ± 332, 3183 ± 365, 3285 ± 347, and 3125 ± 306 g, respectively. The four groups varied significantly in infant birth weight (χ^2^ = 3.885, *p* < 0.05).

Linear regression models were applied to explore linear relationships between different fluid intake types, participants’ body compositions, and their infants’ birth weights. To begin with, fluid intake and body-composition variables were input into a linear regression model (Model 1). The method of backward elimination was used in the model fitting. A collinearity diagnosis and variable adjustment were applied to improve the goodness of fit and stability of the model. As a result, two models were fitted to the data: Model 1 presented a linear regression analysis in which fluid intake and body-composition variables were entered, and Model 2 was the model after adjustment. Due to the correlation between TFI and plain water intake (conducted using the Spearman test, r = 0.866, *p* < 0.001), the variable of TFI was not included in Model 1. Potential confounding factors were selected and included in the two regression models based on previous analyses, including maternal weight and BMI.

The analysis showed linear relationships between plain water intake, basal metabolic rate, and infant birth weight (SE = 0.153, *p* < 0.05; SE = 0.076, *p* < 0.05). There were no linear relationships between dairy product intake, SSB intake, skeletal muscle, body water content, percent of body fat, or infant birth weight (all *p* > 0.05) (Table 7).

## 4. Discussion

Our present study investigated TFI levels and types of pregnant women during their third trimester. Maternal body compositions were also measured. Related urine indicators were collected and assessed to evaluate their hydration status. Furthermore, the relationships between different types of fluid intake, maternal body compositions, and infant birth weights were analyzed. The results indicated that most pregnant women in their third trimester had insufficient TFI, with only 12.1% of participants meeting the Chinese water AI level (1.7 L/day for pregnant women). A study performed on 583 Chinese pregnant women revealed that TFI levels in the third trimester were 1446 mL, lower than the results obtained from our study (1574 mL) [50]. Notably, the retrospective questionnaire was used in the previous one, while a more accurate 7-day, 24 h real-time fluid intake record was used in our study. Previous studies revealed that the different methods can result in a deviation of up to 500 mL per day [51,52]. Compared with studies conducted in other countries, TFI levels among Chinese women were lower than those gained in a study among 132 French pregnant women in their third trimester (1937 mL/d) in 2014 [53]. Plain water was the primary type, with the highest intake level in this study accounting for 94.3% of TFI. This was similar to the findings from other studies [25,54].

Urine osmolality is the most accurate indicator to reflect kidney function of concentration and dilution, and it is also the most widely used to evaluate hydration status [55,56]. In this study, the proportion of the participants with a dehydration status was 15.0%, evaluated with urine osmolality. A previous study among pregnant women revealed that over 50% of overweight and obese women in America experienced dehydration status during pregnancy [57]. A study examined 38 pregnant women in West Jakarta during their second trimester; surprisingly, 20 participants had a dehydrated status, resulting in a dehydration rate of 52.6% [58]. It is clear that the hydration status of the participants in our present study is better than that of pregnant women from other countries. Correlation analysis revealed that negative correlations existed between urine osmolality, USG, urine pH, urine acid, and TFIs. There was a positive correlation between TFI, plain water, dairy products, SSBs, and hydration status. This suggests the possibility of urine biomarkers as indicators to reveal fluid intake and hydration status. Urine, an important pathway for water excretion, is crucial in maintaining hydration status. A total of 573 volunteers in Spain, Germany, and Greece participated in an eight-day study, which showed that TFI was negatively associated with urine specific gravity and color [59]. These basic findings were consistent with research showing that biomarkers in morning urine distinguished between high-level and low-level daily water intakes [60].

In our present study, participants with higher TFI levels had higher basal metabolic rates and body water content (χ^2^ = 20.784, *p* < 0.05; χ^2^ = 14.026, *p* < 0.05). This was in line with the result showing that water intake increases fat oxidation. This was under the condition when blood carbohydrate and insulin concentrations were kept stable, and when the fluid type was not caloric beverages, and the hydration status was not altered. Water intake is associated with increased energy consumption in the short term [61]. Another study reported that 500 mL of plain water intake resulted in higher energy consumption than the same volume (500 mL) of saline [62]. The result was also in line with the previous study, which noted the relationship between water intake behaviors and body water content. A study performed on 358 young Spanish women revealed that TFI was positively correlated with body water content (*r* = 0.196, *p* = 0.002; *r* = 0.180, *p* = 0.006). It also suggested that higher TFI was related to lower weight, body fat mass, and waist circumference [63]. Moreover, another study revealed a positive correlation between TFI normalized via body weight and body water content, while there was the inverse with BMI and fat body mass [64]. In a study conducted in America, researchers assessed the body compositions of 440 pregnant women and found that total body water content could be a powerful predictor of deterioration of pre-eclampsia [20]. Thus, adequate fluid intake is required during pregnancy to promote a better body water condition and ensure maternal and fetal health. A linear relationship was found between plain water intake and infant birth weight (*t* = 2.074; *p* < 0.05). This is consistent with a study that found that a low level of fluid intake during pregnancy was a risk factor for low birth weight [31]. A prospective cohort study on 2039 pregnant women in America showed that, after confounding adjustment, infant birth weight increased with the increase in the maternal TFI level during pregnancy [65]. Infant birth weight correlated with body adipose tissue increases in early pregnancy [18]. Fat-free mass and total body water content have been shown to be associated with birth weight, and total body water gain was positively linked to birth weight from studies conducted in different countries [13,66,67,68]. In our study, no linear relationship existed between body water content and infant birth weight (*p* > 0.05). It is worth noting that maternal dietary and nutritional statuses have an essential effect on the programming of the body [69]. Maternal and infant health may be influenced by these confounding factors in this study, and the association and mechanism are still unclear. Meanwhile, no linear relationship existed between the percent of body fat and infant birth weight (*p* > 0.05). This was similar to a previous study indicating that infant birth weight was positively correlated with maternal fat-free mass [70]. These findings suggest that increasing body fat mass during pregnancy may not protect infants from low birth weight to a certain extent. The results showed that participants with higher basal metabolic rates were associated with higher infant birth weight. A previous study showed that extra energy is in demand during pregnancy for supporting fetal development, maternal tissue increase, and maternal energy-metabolism change [71].

Our study has some strengths and limitations. Regarding the strengths, this is the first Chinese study to explore TFI, maternal body compositions in the third trimester during pregnancy, and their relationships with infant birth weight in China. Additionally, detailed data on fluid intake types were collected in this study. Thus, this led to further analysis of the correlations between different types of fluid and infant birth weight. What is more, a 7-day, 24 h fluid intake record in real-time was performed, which significantly reduced the inaccuracy associated with retrospective recording. In addition, multiple body composition indicators during pregnancy were detected, and their relationships with infant birth weight were analyzed. Last but not least, potential confounders have been part of our models, such as BMI and weight. The robustness of our results was enhanced by this comprehensive analysis. However, there are also notable limitations. First, only morning urine osmolality was obtained, lacking the 24 h urine osmolality. As circadian fluctuations influence urine biomarkers, this may result in some bias in evaluating hydration status throughout the day [72]. Secondly, only the TFI levels of participants were explored in our present study because of the unavailability of data on water intake levels from food. The result cannot be applied comprehensively to reflect pregnant women’s total water intake level. Additionally, only infant birth weight was measured, while height, head circumference, and hip circumference were not. This leads to a lack of comprehensive assessment of infant growth status. Therefore, studies attempting to explore the long-term effects of TFI and hydration status during pregnancy and lactation on maternal and child health are needed in the future.

## 5. Conclusions

Water intake was insufficient among pregnant women during the third trimester, with only 12.1% of participants meeting the recommended adequate fluid intake for pregnant women living in China (1.7 L). The participants displayed a poor hydration status. There may be potential relationships between plain water intake, basal metabolic rate, and infant birth weight. Further studies should focus on the long-term dynamic monitoring of fluid intake during pregnancy and postnatal to effectively analyze the influence on maternal and child health.

## Figures and Tables

**Table 1:** Indicators and different time points for collection in this study.

	The Third Trimester of Pregnancy							Delivery
	Day 1	Day 2	Day 3	Day 4	Day 5	Day 6	Day 7	
Individual information	√ *							
Physical measurements	√							
Body-composition measurements	√							
7-day 24 h fluid intake record	√	√	√	√	√	√	√	
Fasting blood samples				√				
Morning urine samples and relevant biomarkers				√				
Temperature and humidity	√	√	√	√	√	√	√	
Infant birth weight								√

Note: *, means the data was collected on that day.

**Table 2:** Thresholds for determining hydration status based on urine osmolality.

Definition of Hydration Status	Urine Osmolality Values (mOsm/kg)
Dehydrated status	urine osmolality > 800 [47,48]
Normal hydrated status	500 < urine osmolality ≤ 800 [4]
Optimal hydrated status	urine osmolality ≤ 500 [49]

**Table 3:** Characteristics of participants.

	LFI_1_	LFI_2_	HFI_1_	HFI_2_	Total	*p*
	(n = 95)	(n = 95)	(n = 95)	(n = 95)	(n = 380)	
Age (year) ^a^	30.0 (26.0~32.0)	29.0 (26.5~31.0)	28.0 (26.0~31.0)	28.0 (26.0~31.5)	29.0 (26.0~32.0)	0.791
Height (cm) ^a^	156.5 (154.3~160.0)	156.0 (153.0~159.0)	157. (153.0~161.0)	158.0 (154.8~161.0)	156.5 (153.5~160.0)	0.101
Weight (kg) ^a^	60.5 (54.2~65.8)	60.7 (54.2~67.8)	58.5 (54.1~63.2)	58.4 (55.1~62.6)	59.3 (54.2~65.0)	0.187
BMI (kg/m^2^) ^a^	24.4 (22.9~26.9) ^b^	24.6 (22.6~26.9) ^c^	23.8 (22.4~26.4) ^b^	23.2 (22.1~25.1) ^d^	24.1 (22.4~26.3)	0.023 *
Blood pressure ^a^						
Systolic (mmHg) ^a^	117 (107~123)	115 (108~122)	117 (110~123)	115 (105~120)	115 (108~122)	0.308
Diastolic (mmHg) ^a^	74 (67~79)	75 (68~80)	74 (69~77)	71 (66~77)	74 (68~78)	0.174
Blood glucose (mmol/L) ^a^	3.7 (3.4~4.3)	3.7 (3.4~4.2)	3.5 (3.3~4.2)	3.7 (3.3~4.7)	3.7 (3.3~4.4)	0.166

Note: ^a^ Values were shown as median (Q_1_~Q_3_) and compared using the Kruskal–Wallis test, whose degrees of freedom (df) were 3. *, means the *p*-value was less than 0.05, indicating that there was a significant difference. ^(b–d)^: The same letter indicates no statistically significant differences between the two groups; different letters indicate significant differences for *p* < 0.05. BMI: body mass index. LFI_1_: low fluid intake 1; LFI_2_: low fluid intake 2; HFI_1_: high fluid intake 1; HFI_2_: high fluid intake 2.

**Table 4:** Composition of fluid intake of participants with different TFI levels.

	LFI_1_	LFI_2_	HFI_1_	HFI_2_	Total	*p*
	(n = 95)	(n = 95)	(n = 95)	(n = 95)	(n = 380)	
Daily TFI (mL) ^a^	1421 (1374~1456) ^c^	1534 (1511~1557) ^d^	1607 (1590~1623) ^e^	1697 (1600~1747) ^f^	1574 (1488~1641)	<0.001 *
Percentage meeting Chinese fluid AI level (%) ^b^	0 (0.0) ^c^	0 (0.0) ^c^	0 (0.0) ^c^	46 (48.4) ^d^	46 (12.1)	<0.001 *
TFI sources						
Plain water						
Amount (mL) ^a^	1349 (1304~1391) ^c^	1447 (1412~1478) ^d^	1506 (1455~1551) ^e^	1579 (1544~1631) ^f^	1467 (1397~1549)	<0.001 *
Percent (%) ^a^	95.3 (93.1~96.9) ^c^	94.4 (92.1~96.2) ^d^	93.5 (91.4~96.2) ^e^	93.6 (90.5~95.4) ^e^	94.3 (91.6~96.2)	0.002 *
Dairy products						
Amount (mL) ^a^	47 (27~79) ^c^	57 (34~86) ^d^	63 (36~88) ^d^	70 (43~94) ^e^	59 (33~89)	0.044 *
Percent (%) ^a^	4.0 (2.0~5.8)	3.9 (2.3~6.2)	4.2 (2.3~6.9)	4.6 (2.6~6.0)	4.1 (2.3~6.0)	0.783
SSBs						
Amount (mL) ^a^	0 (0~31) ^c^	21 (0~61) ^d^	27 (0~69) ^d^	43 (0~83) ^e^	24 (0~64)	<0.001 *
Percent (%) ^a^	0.0 (0.0~2.3) ^c^	1.4 (0.0~3.9) ^d^	1.7 (0.0~4.3) ^e^	2.5 (0.0~4.7) ^f^	1.5 (0.0~3.9)	0.001 *

Note: ^a^ Values presented as median (Q_1_~Q_3_) and compared using the Kruskal–Wallis test, whose degrees of freedom (df) were 3; ^b^ values represent n (percentage) which were compared using the chi-squared test. * values mean significant differences existed as the *p*-value was less than 0.05. ^(c–f)^: The same letter indicates no statistically significant differences between the two groups; different letters indicate significant differences for *p* < 0.05. AI represents recommendations for adequate intake levels. The AI recommendation for TFI levels for pregnant women set by the Chinese Nutrition Society is 1.7 L per day. LFI_1_: low fluid intake 1; LFI_2_: low fluid intake 2; HFI_1_: high fluid intake 1; and HFI_2_: high fluid intake 2. TFI: total fluid intake; SSBs: sugar-sweetened beverages.

**Table 5:** Urine indexes for participants with different TFI levels.

	LFI_1_	LFI_2_	HFI_1_	HFI_2_	Total	*p*
	(n = 95)	(n = 95)	(n = 95)	(n = 95)	(n = 380)	
Urine osmolality (mOsm/kg) ^a^	689 (597~767) ^c^	672 (569~753) ^c^	664 (560~763) ^c^	562 (356~733) ^d^	666 (554~763)	<0.001 *
Hydration status						
Optimal hydrated status (n,%) ^b^	6 (6.3%)	4 (4.2%)	4 (4.2%)	15 (15.8%)	29 (7.6%)	
Normal hydrated status (n,%) ^b^	53 (55.8%)	79 (83.2%)	88 (92.6%)	74 (77.9%)	294 (77.4%)	<0.001 *
Dehydrated status (n,%) ^b^	36 (37.9%)	12 (12.6%)	3 (3.2%)	6 (6.3%)	57 (15.0%)	
Urine specific gravity (USG) ^a^	1.020 (1.015~1.026) ^c^	1.020 (1.015~1.023) ^c^	1.018 (1.010~1.021) ^c^	1.010 (1.010~1.020) ^d^	1.015 (1.010~1.023)	<0.001 *
Urine pH ^a^	6.0 (5.0~6.5) ^c^	6.0 (5.0~6.5) ^d^	6.0 (5.0~6.5) ^c^	6.0 (5.9~7.0) ^e^	6.0 (5.5~6.5)	0.020 *
Urea (mmol/L) ^a^	4.2 (3.5~5.0)	3.9 (3.4~4.6)	4.0 (3.3~4.7)	3.9 (3.3~4.4)	4.0 (3.3~4.7)	0.115
Urine creatinine (mmol/L) ^a^	59.2 (52.3~66.3) ^c^	58.5 (51.8~63.7) ^d^	56.6 (52.3~63.7) ^d^	54.6 (52.6~58.0) ^e^	56.6 (52.2~56.3)	0.014 *
Uric acid (mmol/L) ^a^	277 (229~321) ^c^	276 (231~305) ^c^	267 (221~3.7) ^c^	237 (209~281) ^d^	265 (223~307)	0.003 *

Note: ^a^ Values presented as median (Q_1_~Q_3_) and compared using the Kruskal–Wallis test, whose degrees of freedom (df) were 3; ^b^ Values presented as n (percentage) and compared using the chi-square test. * Values mean significant differences existed, as a *p*-value of less than 0.05 was considered significant. ^(c–e)^: The same letter indicates no statistically significant differences between the two groups; different letters indicate significant differences for *p* < 0.05. LFI_1_: low fluid intake 1; LFI_2_: low fluid intake 2; HFI_1_: high fluid intake 1; and HFI_2_: high fluid intake 2.

**Table 6:** Body compositions of participants with different TFI levels.

	LFI_1_	LFI_2_	HFI_1_	HFI_2_	Total	*p*
	(n = 95)	(n = 95)	(n = 95)	(n = 95)	(n = 380)	
Skeletal muscle (kg) ^a^	43.0 (39.4~44.9)	50.4 (39.8~45.0)	49.2 (40.3~45.0)	50.8 (41.0~45.1)	49.4 (40.1~45.0)	0.262
Bone mineral content (kg) ^a^	2.1 (2.0~2.2)	2.1 (2.0~2.3)	2.1 (2.0~2.2)	2.1 (2.0~2.3)	2.1 (2.0~2.3)	0.572
Basal metabolic rate (kcal) ^a^	2173 (1972~2302) ^b^	2202 (2108~2357) ^c^	2253 (2086~2395) ^d^	2314 (2153~2515) ^e^	2241 (2086~2394)	<0.001 *
Percent body fat (%) ^a^	27.4 (25.5~29.2)	28.3 (26.7~29.9)	28.0 (24.9~29.9)	28.6 (26.5~30.1)	28.1 (25.8~29.9)	0.262
Body water content (%) ^a^	48.6 (46.4~52.0) ^b^	50.4 (46.8~53.5) ^c^	49.2 (46.9~53.2) ^b^	50.8 (48.0~54.2) ^d^	49.4 (46.9~53.2)	0.003 *

Note: ^a^ Values presented as median (Q_1_~Q_3_) and compared using the Kruskal–Wallis test, whose degrees of freedom (df) were 3. * values mean significant differences existed, as a *p*-value of less than 0.05 was considered significant. ^(b–e)^: The same letter indicates no statistically significant differences between the two groups; different letters indicate significant differences for *p* < 0.05. LFI_1_: low fluid intake 1; LFI_2_: low fluid intake 2; HFI_1_: high fluid intake 1; and HFI_2_: high fluid intake 2.

**Table 7:** Associations between fluid intake levels and body composition with infant birth weight among participants.

Variables	Dependent Variable	Unstandardized Coefficient		Standardized Coefficient	*p*	VIF
		*B*	SE	*β*		
Model 1						
Plain water (mL)	Infant birth weight	0.384	0.160	0.129	0.017 *	1.113
Dairy products (mL)		0.400	0.469	0.045	0.395	1.056
SSBs (mL)		0.007	0.394	0.001	0.985	1.037
Skeletal muscle (kg)		0.693	1.705	0.006	0.913	1.026
Bone mineral content (kg)		94.089	81.539	0.063	0.249	1.118
Body water content (%)		2.999	3.250	0.049	0.357	1.427
Percent body fat (%)		0.078	1.705	0002	0.964	1.022
Basal metabolic rate (kcal)		0.194	0.090	0.131	0.032 *	1.085
Model 2						
Plain water (mL)	Infant birth weight	0.395	0.153	0.133	0.011 *	1.038
Basal metabolic rate (kcal)		0.208	0.076	0.140	0.007 *	1.038

Note: Linear regression models were applied to explore the linear relationship between different types of maternal fluid intake and infant birth weight. *, A *p*-value of less than 0.05 was considered significant. Infant birth weight was the dependent variable in the two models. B: unstandardized coefficients; SE: standard error of the coefficients; β: standardized coefficients; VIF: variance inflation factors; and SSBs: sugar-sweetened beverages.

## Data Availability

The data can be obtained from the corresponding authors on reasonable request. As the data was currently explored and further analyzed, it will not be disclosed publicly at this stage.

## Abbreviations

AI	Adequate intake
BMI	Body mass index
SSB	Sugar-sweetened beverage
TFI	Total fluid intake
USG	Urine specific gravity
